# Automatic analysis (aa): efficient neuroimaging workflows and parallel processing using Matlab and XML

**DOI:** 10.3389/fninf.2014.00090

**Published:** 2015-01-15

**Authors:** Rhodri Cusack, Alejandro Vicente-Grabovetsky, Daniel J. Mitchell, Conor J. Wild, Tibor Auer, Annika C. Linke, Jonathan E. Peelle

**Affiliations:** ^1^Brain and Mind Institute, Western UniversityLondon, ON, Canada; ^2^Donders Institute for Brain, Cognition and BehaviourNijmegen, Netherlands; ^3^MRC Cognition and Brain Sciences UnitCambridge, UK; ^4^Department of Otolaryngology, Washington University in St. LouisSt. Louis, MO, USA

**Keywords:** neuroimaging, functional magnetic resonance imaging (fMRI), diffusion tensor imaging (DTI), diffusion weighted imaging (DWI), multi-voxel pattern analysis (MVPA), software, pipeline

## Abstract

Recent years have seen neuroimaging data sets becoming richer, with larger cohorts of participants, a greater variety of acquisition techniques, and increasingly complex analyses. These advances have made data analysis pipelines complicated to set up and run (increasing the risk of human error) and time consuming to execute (restricting what analyses are attempted). Here we present an open-source framework, automatic analysis (*aa*), to address these concerns. Human efficiency is increased by making code modular and reusable, and managing its execution with a processing engine that tracks what has been completed and what needs to be (re)done. Analysis is accelerated by optional parallel processing of independent tasks on cluster or cloud computing resources. A pipeline comprises a series of modules that each perform a specific task. The processing engine keeps track of the data, calculating a map of upstream and downstream dependencies for each module. Existing modules are available for many analysis tasks, such as SPM-based fMRI preprocessing, individual and group level statistics, voxel-based morphometry, tractography, and multi-voxel pattern analyses (MVPA). However, *aa* also allows for full customization, and encourages efficient management of code: new modules may be written with only a small code overhead. *aa* has been used by more than 50 researchers in hundreds of neuroimaging studies comprising thousands of subjects. It has been found to be robust, fast, and efficient, for simple-single subject studies up to multimodal pipelines on hundreds of subjects. It is attractive to both novice and experienced users. *aa* can reduce the amount of time neuroimaging laboratories spend performing analyses and reduce errors, expanding the range of scientific questions it is practical to address.

## The need for efficient workflows

The last two decades have seen enormous growth in the use of magnetic resonance imaging (MRI) as a tool to understand brain function, and in the size and complexity of the datasets acquired. The number of participants in individual studies has grown for many reasons, including: the increasing availability of MRI scanners; a move from fixed- to random-effects designs (Friston et al., 1999; Mumford and Nichols, 2008); a demand for greater replication in neuroimaging (“The dilemma of weak neuroimaging papers,” http://www.danielbor.com/dilemma-weak-neuroimaging); the need to overcome statistical noise in studies of individual differences, genetics, aging, development or disease; large scale investments such as the Human Connectome Project (Van Essen et al., 2012), Alzheimer's Disease Neuroimaging Initiative (Mueller et al., 2005) or Cambridge Centre for Aging and Neuroscience (http://www.cam-can.org); and a growth in open data sharing (Van Horn et al., 2001; Biswal et al., 2010; Poldrack et al., 2013; http://www.xnat.org).

Furthermore, the neuroimaging data acquired from each participant have become richer. Whereas in the past, researchers frequently collected data using a single method, many now acquire diverse MRI protocols, including structural (e.g., T1, T2, PD), functional (echoplanar imaging; EPI), connectivity (diffusion-weighted imaging; DWI), fieldmaps (multi-echo; gradient echo) and myelination (magnetization transfer ratio; MTR) measurements in single studies. Accelerated sequences using parallel imaging (SENSE, GRAPPA, and multiband EPI) have allowed for finer temporal or spatial resolution and increased the size of datasets by up to an order of magnitude.

Alongside the increasing quantity of data, the palette of analysis methods has also grown. In functional MRI (fMRI), in addition to the standard preprocessing stages of motion correction, slice-timing correction, warping-to-template (normalization) and smoothing, denoising is now possible using tools based upon independent components analysis (Calhoun et al., 2009; Kundu et al., 2012; http://fsl.fmrib.ox.ac.uk/fslcourse/graduate/ica_prac/artdata/dim33.ica/report; http://fsl.fmrib.ox.ac.uk/fsl/fslwiki/FIX), modeling of noise components (Kay et al., 2013), and image rejection (Power et al., 2012). Statistical analyses are now often conducted both using standard univariate methods and multi-voxel pattern analysis (MVPA) (Haynes and Rees, 2006; Kriegeskorte et al., 2006; Norman et al., 2006). Brain structure is often analyzed using voxel- (Ashburner, 2009) and surface-based (Winkler et al., 2012) morphometry, and gyrification indices (Schaer et al., 2008). Registration between individuals can use relatively low-dimensional warping to a template, or higher dimensional registration (Ashburner, 2007, 2009). Diffusion data can be analyzed with probabilistic or deterministic methods, by summarizing parameters such as the fractional anisotropy (FA) on a skeleton (Smith et al., 2006) or by tracing tracts (Behrens et al., 2007). In addition to the sheer number of useful analysis methods now available, many methods are highly computationally intensive, such as searchlight MVPA (Kriegeskorte et al., 2006), probabilistic tractography, and high-dimensional image warping (Ashburner, 2007). Implementing these complementary approaches commonly requires a combination of software packages, which follow diverse concepts and may even use different file formats. The integration of results from these different software packages (e.g., using fMRI activation clusters as seeds for diffusion tractography) further increases the complexity of an analysis workflow.

The increasing quantity of raw data and greater number of computationally intensive analysis methods have led to two challenges. The first is an increase in the complexity of the workflows required: There are a greater number of individual “chunks” of processing, and more complex dependencies between these chunks. Furthermore, even the best-run neuroimaging study does not always proceed exactly according to plan, and there are often idiosyncrasies that result from technical glitches, operator error, or participant non-compliance. Manual intervention in this complex workflow leads to the potential for human error.

The second challenge is an increase in computation time per study. Many neuroimagers are already stretched by the need to become multidisciplinary experts in the physics of neuroimaging, the mathematics for analysis, the psychology of cognitive function, and the biology of the brain. They do not all necessarily relish the additional challenge of becoming a programmer and computer scientist so that they can make the most efficient use of computing resources.

The many stages of analysis required to draw conclusions from MRI data were once almost universally accomplished using point-and-click interfaces, a practice many continue. However, as the field matures, this sort of “manual” analysis is becoming increasingly impractical and unattractive. Here, we present a software package, automatic analysis (*aa*) (http://automaticanalysis.org), which provides a simple but flexible way to specify complex workflows, keep track of what needs to be done, and facilitate parallel computing. *aa* is engineered so that even when used by a “lazy” operator precise records are kept. It is easily extendable, and code naturally becomes re-useable and shareable.

## Existing software

Once the decision is made to use a processing pipeline, there are a number of options. Although the best solution depends a great deal on individual preferences and priorities, we have engineered *aa* to fill needs not met by other processing pipelines.

Neuroimaging benefits enormously from a dynamic software development community, with new analysis tools frequently disseminated by large teams. However, these packages focus primarily on implementing specific tools, rather than managing efficient workflows. *aa* provides access to many (though not all) functions in the major neuroimaging packages of SPM, FSL, and Freesurfer; other tools such as the Advanced Normalization Tools (ANTs); and our own implementation of searchlight- or ROI- based MVPA. In addition, although not discussed in this manuscript, it also includes growing support for other modalities including MEG, EEG, and ECoG.

## Design goals

### Efficient and easy-to-read specification of complex pipelines

As neuroimaging pipelines become increasingly complicated, it becomes important to develop elegant ways of describing them. With *aa*, we aimed to separate a high-level description of what needs to be done (e.g., motion correction followed by slice-timing correction) from the individual parameters that control each stage. Furthermore, wherever possible, sensible default values are available for each stage, so that an analysis can be specified as leanly and efficiently as possible, without the need to re-invent the wheel each time. We make extensive use of XML markup language to provide easy-to-read descriptions of tasklists (i.e., the list of processing stages) and settings.

### Modular design

To make it easier to identify the code that is responsible for a given task, and to facilitate parallel computing, each stage of processing is described by an encapsulated “module.”

### Separation of method and data

A separation is enforced between the algorithms that should be applied and the data (i.e., participants and sessions) on which they should operate. This separation ensures that modules are re-useable: once written in the context of one analysis, modules may usually be re-used without modification in another analysis of different data.

### Only do what needs to be done

Modules are never called directly by the user; instead, their execution is handled by the *aa* scheduling engine (*aa_doprocessing*). The scheduling engine identifies whether a module has already been run on a given piece of data, and whether the inputs to a module have changed (e.g., a subject has been added) since it was last run. If a module has already been run, it is not repeated. Although simple, checking for completed stages provides three important practical benefits. First, it saves computational resources. Second, it makes debugging quicker: If an analysis crashes partway through, then the next time it is re-run, all of the stages that lead up to the crashing stage will not be executed. Third, it stops the user from needing to “comment out” lines that have already completed when rerunning just one later part again. As a result, in practice the final *aa* script will typically recreate an analysis in its entirety.

Checking for previously-completed stages also facilitates complex pipelines with multiple analysis pathways. For example, in the case where all processing stages save one are identical (e.g., to compare preprocessing with and without slice-timing correction), *aa* can be informed about a branched tasklist and re-use inputs that are common to both branches.

### Facilitate parallel processing

As analyses become more computationally intensive, being able to easily accelerate them across a cluster of machines is increasingly important. Often, execution time determines what analyses a user can bear. For example, even if an analysis runs in a single-threaded manner in a practical amount of time (say 5 days), a user will be highly discouraged from running it again to fix some small issue.

*aa* uses coarse-grained parallelization, meaning that where possible, multiple modules, different EPI sessions, subjects, or even analyses (e.g., groups of searchlights in an MVPA analysis for a single module) are run in parallel. Modules themselves are not written differently for parallel or single-threaded execution: parallelization is achieved entirely in the scheduling engine (although individual modules can in principle be parallelized at a finer-grained level).

### Keep track of what has happened

A precise record of everything that has happened in an *aa* analysis is saved and can be referred to in the future. It is stored as a Matlab structure, which can be read back in to recreate the analysis, or probed for parameter settings.

### Diagnostics and quality control

One of the drawbacks of batch analysis is that a user may be tempted to only look at the final results, and not inspect the data at each stage of processing. However, complex analysis pipelines can fail in a greater number of ways than simpler pipelines. Some failures can be obvious (e.g., activation outside the brain due to imperfect registration), while others are harder to track down (e.g., weaker group activation detected due to high between-subject variability caused by motion). Consequently, inspection of data is as important as ever. Several existing solutions generate some diagnostic data during the analysis (e.g., FSL's FEAT Pre-stats and Registration reports); however, the information provided is limited, sometimes complicated to reach, and almost never submitted to between-subject analysis (important for the measurement of between-subject variance and outlier detection).

To address this problem, many *aa* modules create diagnostic results (e.g., plots of motions to be corrected, registration overlays, thresholded statistical parameter maps for first-level contrasts). In addition, *aa* also implements various quality control tools (mostly SPM- and FSL-based). A dedicated module for low-level quality control (*tsdiffana*) is also bundled with *aa*, which—thanks to the flexible modular concept—can be employed before or after various stages or even multiple times, which allows a user to follow how the data change during the analysis. Conveniently, these diagnostic results are collected into a central place in a multi-level fashion, allowing a user to browse both vertically (within-subject) and horizontally (between-subject). Where applicable (e.g., motion correction), between-subject visual comparison and/or statistics are also provided.

## System and software requirements

- *aa* is developed in a ^*^nix environment and actively used on machines running Ubunto, RedHat, and Mac OS X. It is not currently supported on Windows.
- *aa* is Matlab-based and requires a base installation of Matlab. Some functions may require additional toolboxes; for example the Image Processing Toolbox. In general, though, *aa* is written with the goal of minimizing use of Matlab toolboxes by using versions of functions included in the base Matlab installation or by recreating these functions.
- As a processing pipeline, *aa* does not include external software (such as SPM, FSL, etc.), which must be installed separately and placed in a user's path.

## Software architecture

This manuscript describes *aa* version 4.2. Not all components apply to earlier versions. The latest version is available from: http://automaticanalysis.org/getting-started/download-installation/. Here, we describe the components in the order a typical user might encounter them, providing a description of each and the motivation for the architecture. The earlier topics will be needed by any *aa* user, while the later ones are likely to be of more interest to experienced users.

### User script

The core of an *aa* analysis is the user script, which describes what processing should happen, and what data it should be applied to. Almost all analyses will require the user to create a user script in Matlab, typically by modifying an example script (found in the “examples” folder distributed with *aa*). An example user script is shown below:

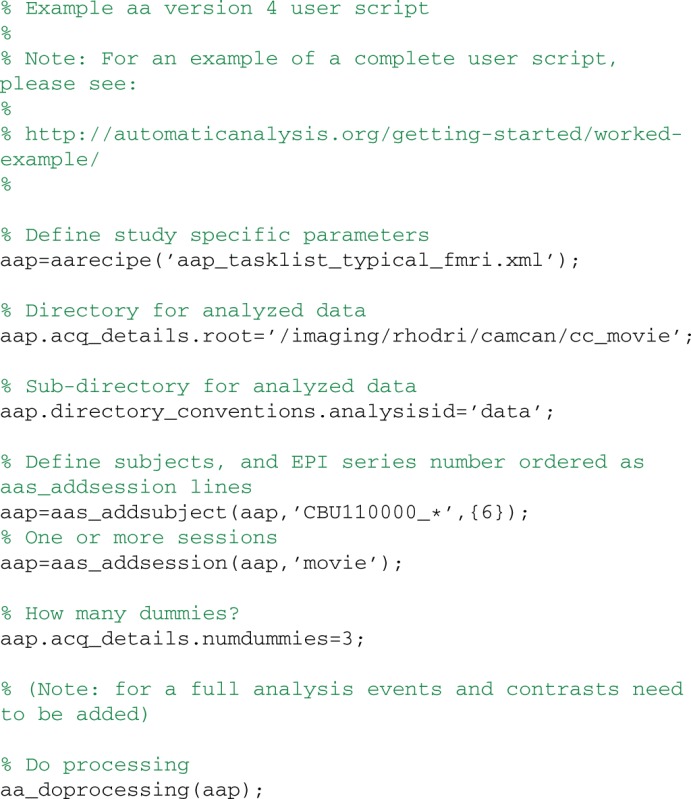



This script executes a typical fMRI processing pipeline (discussed more in the next section) on a single subject (CBU110000) for a single session (imaging series 6, labeled “movie”).

The user script can set parameters, such as output paths, or settings for modules. Here, three dummy scans are specified to be ignored in the analysis by the line:


aap.acq_details.numdummies=3


Note that the entire analysis—comprising the set of tasks to be run and the data they are to be run on—is described in a single structure (the “aap” variable). It is initially constructed by the *aarecipe* command. Because the analysis is fully specified by a single structure (along with the codebase), it is trivial to keep a record of the analysis, or to re-run it at a later date.

### Basic tasklists

The tasklist is an XML format file that describes what should be done. A number of tasklists are available, many of which are useful without modification (Table 1).

**Table 1:** **Example tasklists**.

**Tasklist**	**Purpose**
aap_tasklist_typical_fmri.xml	fMRI preprocessing and first/second level statistics
aap_tasklist_fmri.xml	fMRI preprocessing and first/second level statistics—variant using fieldmaps, realignunwarp.
aap_tasklist_dartelvbm8.xml	VBM with SPM8 and DARTEL
aap_tasklist_diffusion.xml	Diffusion tractography with FSL
aap_tasklist_diffusion2.xml	Nonlinear DTI and DKI
aap_tasklist_freesurfer.xml	Structural processing with Freesurfer

**Figure 1:**
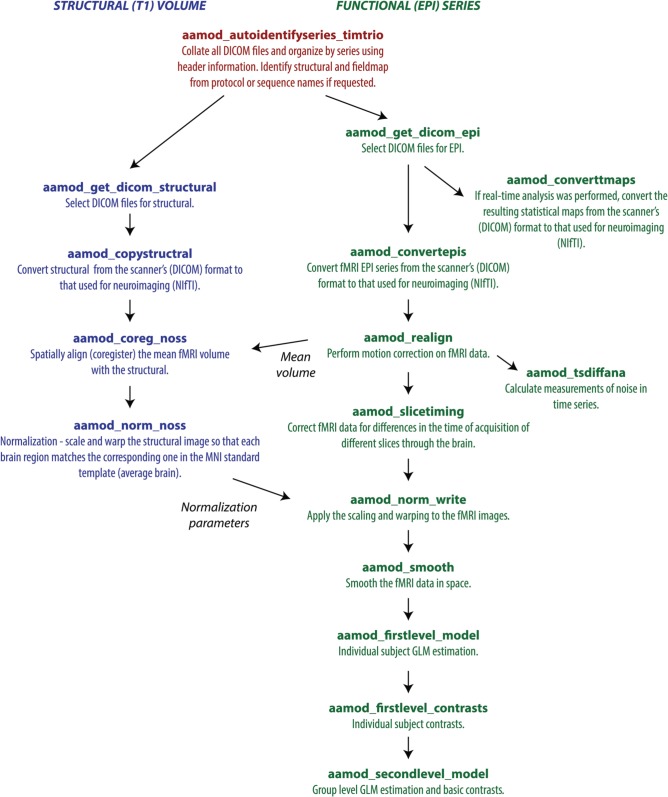
**A typical fMRI pipeline comprising a set of *aa* modules (filenames prefixed with aamod_)**. Blue colors refer to modules processing the structural, green colors processing the EPI, and red are general. This pipeline does preprocessing and first-level (individual) and second-level (group) statistics.

Each tasklist describes a series of modules that should be executed. In the example user script given above, the tasklist specified was *aap_tasklist _typical_fmri.xml*. Figure 1 shows the processing that will be run for this tasklist. Note that a subject's structural (T1) and fMRI (EPI) data go through a number of processing stages, and some modules operate on the data together. The XML code that underlies this tasklist is below.

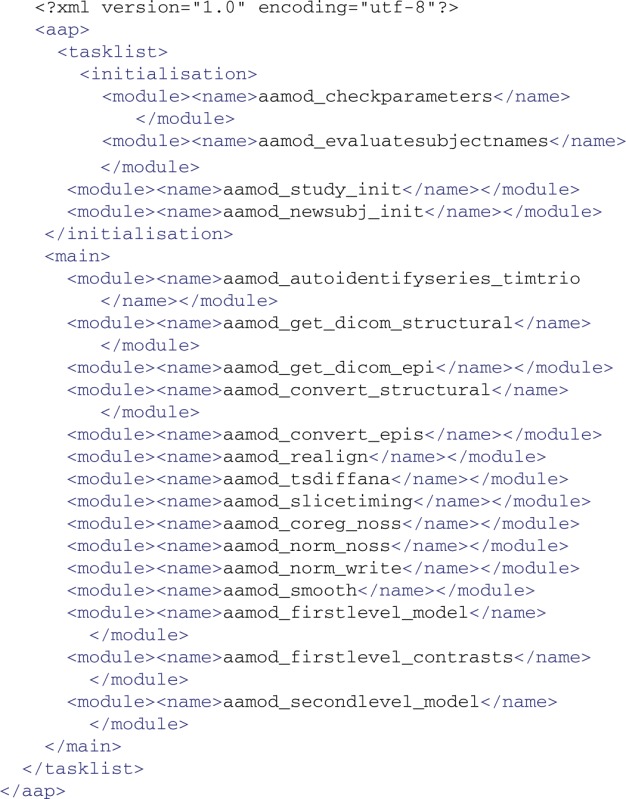



There are two sections to this simple tasklist. The “initialisation”^1^ modules are run every time, for tasks such as checking the input parameters, or expanding wildcards in the subject names. The “main” modules are only run once on each piece of data, unless an explicit re-run is requested.

Note also that the dependencies (that is, which pieces of data act as the input to each module) are not usually explicitly specified in the tasklist. Instead, the pipeline is automatically connected up at the start of processing using information in each module's interface. This simplifies specification of tasklists, and allows modules to be reordered with reduced potential for error. The dependencies are reported at the start of an analysis.

### Output file structure

**Figure 2:**
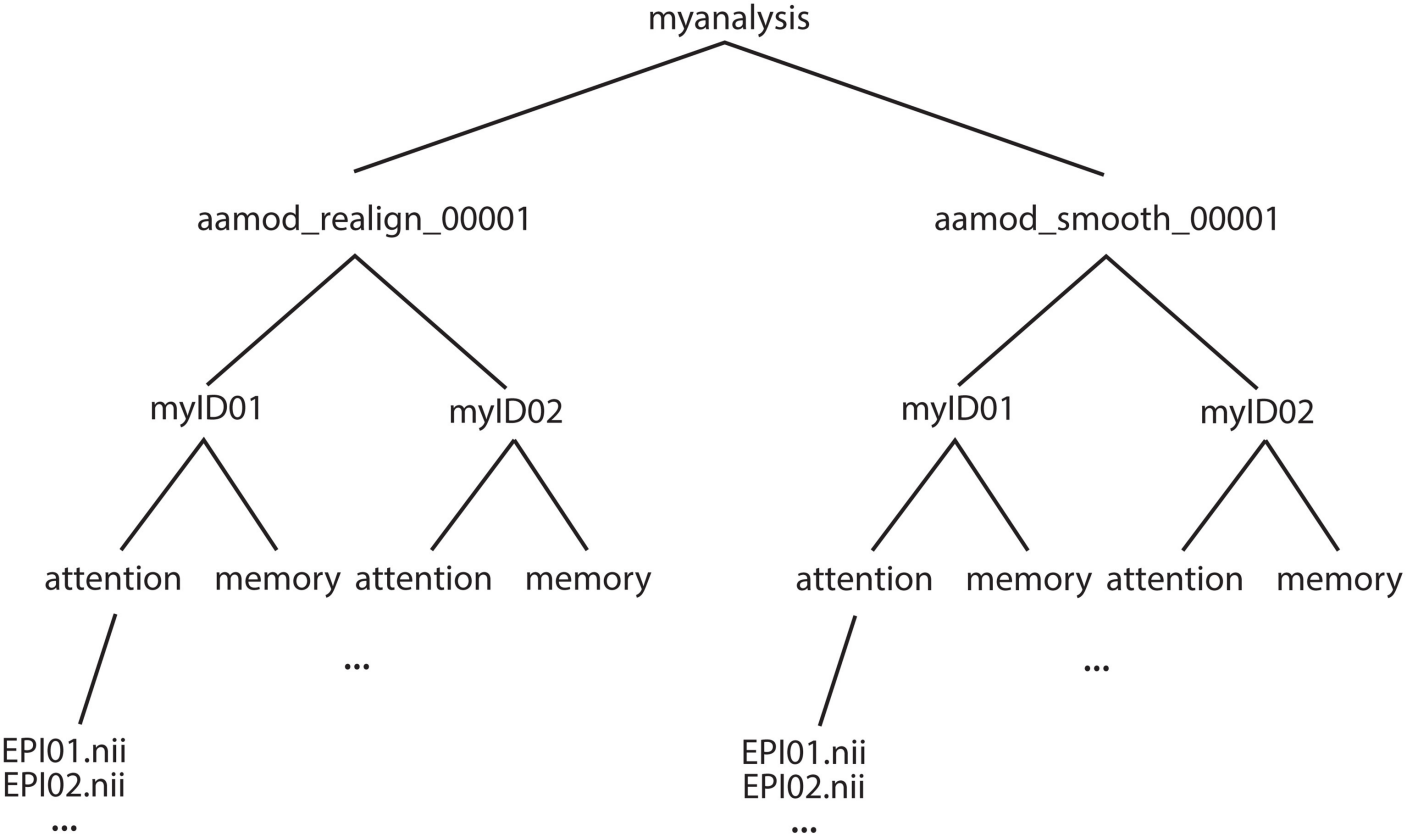
**Example file structure for *aa* output**. Each analysis comprises output directories organized by processing stage (here, for example, realignment and smoothing) which are then each subdivided by subject, then session.

The example of an output file tree for an *aa* analysis is shown in Figure 2. The path to which this structure gets written is determined by the *aa* setting


aap.acq_details.root=’/imaging/rhodri/mypath’;


The name of the directory for the analysis is specified in:


aap.directory_conventions.analysisid=’myanalysis’;


Each module operates on data stored in a separate directory (e.g., *aamod_realign_00001, aamod_smooth_00001*). This differs from the conventions with packages such as SPM where all analysis stages are written to a single directory, often with different prefixes or suffixes to distinguish the stages. There are a number of practical benefits to *aa*'s directory separation. First, it reduces the number of files within subdirectories, which makes them more manageable, particularly for fMRI or DTI with a 3D data format (e.g., one image per timepoint). Second, it makes it easier to see at a glance what processing has happened, and to find a file when browsing. Finally, it makes maintenance easier when, for example, a user wishes to delete intermediate stages of analysis to save disk space.

These ease-of-use and aesthetic advantages come along with more fundamental benefits. Partitioning the workspace of modules into separate directories facilitates the encapsulation of data. The *aa* engine is responsible for putting a module's input data into the directory in which it will execute. If a module does not request a piece of data, it will not be there, and it cannot accidentally be used. Similarly, the *aa* engine is responsible for picking up outputs and passing them along the pipeline. If a module does not explicitly declare an output, it will not be passed. Thus, directory separation allows the *aa* scheduling engine to maintain tight control of data dependencies. This has a number of benefits. It permits parallel processing with a reduced potential for conflicts due to unexpected module behaviors. When executing on a cluster, data transfer demands are reduced as a compute node does not need to receive the whole analysis, but only the specific data it is working on. Finally, the one-directory-per-module structure facilitates branched tasklists, where an analysis forks, and is continued in two different ways (e.g., with a smoothing kernel of 8 or 12 mm).

Here, both modules had the suffix _00001. If either module were present more than once in a tasklist (e.g., *tsdiffana* run before and after a processing stage), this index would be incremented by one for each subsequent entry.

Note that this architecture does not restrict the level at which a module can operate. That is, if data for all sessions and subjects are needed to complete an analysis, they will all be copied to the appropriate directory. However, as this is more often the exception than the rule, on the whole *aa*'s limited copying approach saves bandwidth and reduces opportunities for error.

### Modules

At the heart of every *aa* analysis are the modules. A module performs a single task, such as motion correction or smoothing. Some examples are given in Table 2.

**Table 2:** **Example *aa* modules**.

**Input data sorting**
**aamod_autoidentifyseries_timtrio**
Scan input DICOM files to get series and acquisitions irrespective of filenames, which are typically site-specific. Identify structural and fieldmap series numbers.
**Anatomy**
**Basic structural**
**aamod_get_dicom_structural**
Find all DICOM files corresponding to the structural acquisition.
**aamod_coreg_extended_1**
Coregister an individual's structural to a standard space template using a rigid body transformation, which improves robustness of later normalization stage.
**aamod_norm_noss**
Estimate nonlinear warp that will transform an individual subject's space into a standard template space (SPM normalization).
**aamod_norm_write**
Apply normalization parameters derived from structural to EPIs.
**DARTEL-VBM**
**aamod_biascorrect_segment8**
Run New Segment (introduced in SPM 8) and save bias-corrected image (e.g., for segmenting).
**aamod_segment8**
Tissue class segmentation using New Segment (SPM 8).
**aamod_structuralstats**
Retrieve total tissue class volume and TIV from segmented images.
**aamod_dartel_createtemplate**
Use DARTEL to create a template.
**aamod_dartel_normmni**
Write DARTEL-warped images to MNI space.
**aamod_normalizebytotalgray**
Divide segmented images by total gray matter (proportional scaling).
**aamod_norm_write_dartel**
Apply normalization parameters derived using DARTEL to other modalities (e.g., EPI, contrasts, DWI, ROIs).
**aamod_dartel_denorm**
Transform images in standard MNI space (e.g., atlas labels) into native space based on normalization parameters derived using DARTEL (multimodal).
**Freesurfer surface extraction**
**aamod_freesurfer_initialise**
Prepare for a Freesurfer analysis.
**aamod_freesurfer_deface**
Defaces structural (T1) and produces a mask.
**aamod_freesurfer_deface_apply**
Apply defacing mask to a co-registered image.
**aamod_freesurfer_autorecon_all**
Runs a Freesurfer pipeline with recon-all.
**Anatomical processing from FSL**
**aamod_fsl_FAST**
Use FAST (FSL) for segmentation.
**aamod_fsl_FIRST**
Use FIRST (FSL) to characterize structure shape.
**ANTS software**
**aamod_ANTS_epi2template**
Create transformation matrix for ANTS normalization to study template.
**aamod_ANTS_warp_ROIs**
Apply inverse warp to ROIs.
**aamod_ANTS_warp_cons**
Apply warp to first level contrasts.
**fMRI activation studies**
**fMRI preprocessing**
**aamod_get_dicom_epi**
Find all DICOM files corresponding to the EPI acquisitions.
**aamod_convert_epi**
Convert the DICOM files to NIfTI format. Handles with multi-echo EPI with various weighting schemes.
**aamod_realign**
Perform motion correction with SPM.
**aamod_slicetiming**
Slice timing correction with SPM.
**aamod_coreg_extended_2epi**
Applies to the EPIs the transformation derived from coregistering the structural to a standard-space template (in aamod_coreg_extended_1). Then, fine-tunes the registration of the EPI to the structural with a further coregistration.
**aamod_coreg_noss**
Coregisters structural to mean EPI using SPM.
**aamod_smooth**
Smooth data.
**Distortion correction**
**aamod_fieldmap_undistort**
Use fieldmap (with phase and magnitude) to correct EPI distortions.
**aamod_realignunwarp**
Realign and unwarp from SPM.
**aamod_pewarp_estimate**
**aamod_pewarp_write**
Constrained nonlinear coregistration.
**Statistics**
**aamod_firstlevel_model**
Run first level statistical model. Simple specification of events in user script.
**aamod_firstlevel_contrasts**
Run first level contrasts. Simple specification of contrasts.
**aamod_secondlevel_model**
Run a *t*-test across subjects for every first level contrast.
**aamod_OneWay_ANOVA**
Run repeated measures (across subjects) one-way ANOVA.
**Networks**
**Connectivity matrices**
**aamod_fconnmatrix_seedseed**
Calculate seed-to-seed connectivity matrix from relationship of time-courses across seed regions.
**PPI**
**aamod_vois_extract**
Extract ROI timeseries after first level analysis.
**aamod_ppi_prepare**
Prepare PPI regressors based on ROI timeseries.
**ICA**
**aamod_tensor_ica**
Run individual or group tensor ICA.
**Movie inter-subject correlation analysis**
**aamod_highpassfilter_epi**
High-pass filter fMRI time series using discrete cosine model, like SPM.
**aamod_meantimecourse**
Calculate mean time course for each voxel across subjects.
**aamod_moviecorr_meantimecourse**
Calculate correlation of each subject's timecourse with mean.
**aamod_moviecorr_summary**
Statistics to find which correlations are significant across subjects.
**Diffusion**
**Basic processing**
**aamod_get_dicom_diffusion**
Get a list of all of the DICOM files that correspond to the diffusion series (typically, as identified by aamod_autoidentifyseries_timtrio).
**aamod_convert_diffusion**
Convert diffusion images from DICOM to NIfTI
**aamod_3dto4d_diffusion**
Convert diffusion images from 3D to 4D. The XML file is 'aamod_3dto4d_diffusion.xml' which refers to the matlab file (using mfile_alias) 'aamod_3dto4d.m'.
**aamod_diffusion_eddycorrect**
Use eddy_correct (FSL) to correct image distortions, head movements using affine registration to a reference volume (T2 image).
**aamod_diffusion_extractnodif**
Use FSL to extract the reference(s) image(s) (T2 image with *b*-value of 0), called nodif.
**aamod_bet_diffusion**
Use FSL to extract the brain of the nodif image. Brain extraction toolbox. Its “mfile” is aamod_bet.
**Diffusion tensors**
**aamod_diffusion_dtifit**
Use FSL to fit a diffusion tensor model at each voxel. Note that dtifit is not necessary in order to run probabilistic tractography (which depends on the output of BEDPOSTX).
**aamod_diffusion_dkifit**
Fit diffusion kurtosis parameters using linear model.
**aamod_diffusion_dtinlfit**
Fit diffusion tensor parameters using nonlinear model.
**aamod_coreg_structural2fa**
Coregister structural to diffusion image (dti_FA).
**Probabalistic tractography**
**aamod_unnormalize_seeds**
Use SPM to “unnormalize" the seeds (i.e., apply the inverse matrix to transform the seed (MNI space) to diffusion space).
**aamod_unnormalize_targets**
Use SPM to “unnormalize” the targets (i.e., apply the inverse matrix to transform the targets (MNI space) to diffusion space).
**aamod_diffusion_bedpostx**
Use FSL to apply bedpostx Monte Carlo modeling of PDFs of diffusion parameters.
**aamod_diffusion_probtrackx**
Use FSL to apply probtrackx, which repetitively samples from the distributions on voxel-wise principal diffusion directions, each time computing a streamline through these local samples to generate a probabilistic streamline or a sample from the distribution on the location of the true streamline.
**aamod_diffusion_probtrackxsummarize_indv**
Get the results of probtrackx (diffusion space) of each participant, merge the different splits and transform them to the MNI space.
**aamod_diffusion_probtrackxsummarize_group**
Averages the seed-to-target connectivity images across subjects, which we have used for visualization.
**MVPA**
**aamod_MVPaa_brain_1st**
Runs an MVPA searchlight on a set of beta or *t*-values (typically in native space).
**aamod_MVPaa_brain_SPM**
Convert results from searchlight into NIfTI images readable in SPM.
**aamod_unnormalize_rois**
Set ROIs from standard space into subject space.
**aamod_MVPaa_roi_1st**
Runs an MVPA analysis within an ROI, using a set of beta or *t*-values (typically in native space).

Each module requires two files: an XML interface (e.g., *aamod_smooth.xml)*, and the corresponding Matlab source (e.g., *aamod_smooth.m)*. Occasionally, an interface file may specify a Matlab file with a different name to its source (e.g., *aamod_autoidentifyseries_ge.xml* points to *aamod_autoidentifyseries.m)* using an *mfile_alias*=‘…’ attribute.

One of a module's most important properties, specified in this XML interface, is the “domain” at which it operates. Modules with a domain of “study” are called just once (i.e., a single instance is created each time the module occurs in the processing pipeline). Modules with a domain of “subject” are called once for each subject, while modules with a domain of “session” are called once for each session of each subject. These are the three most common module domains; others include diffusion_session, meg_session, and hyperalignment_searchlight_package. However, new domains can be easily added to the *aa* engine, and user-written modules can make use of new domains.

Instances of a module should restrict their processing to a particular set of input data (i.e., for a given session-domain module, there might be an instance for subject 3, session 2). This instance should take care to only attempt to process this portion of the data, and should never attempt to write data outside its domain (in this example, to another session).

Other important properties of a module are the type of data (e.g., *epi* or *structural*) it requires as an input, and the type of data it produces as an output.

An example interface file, *aamod_smooth.xml*, is shown below.

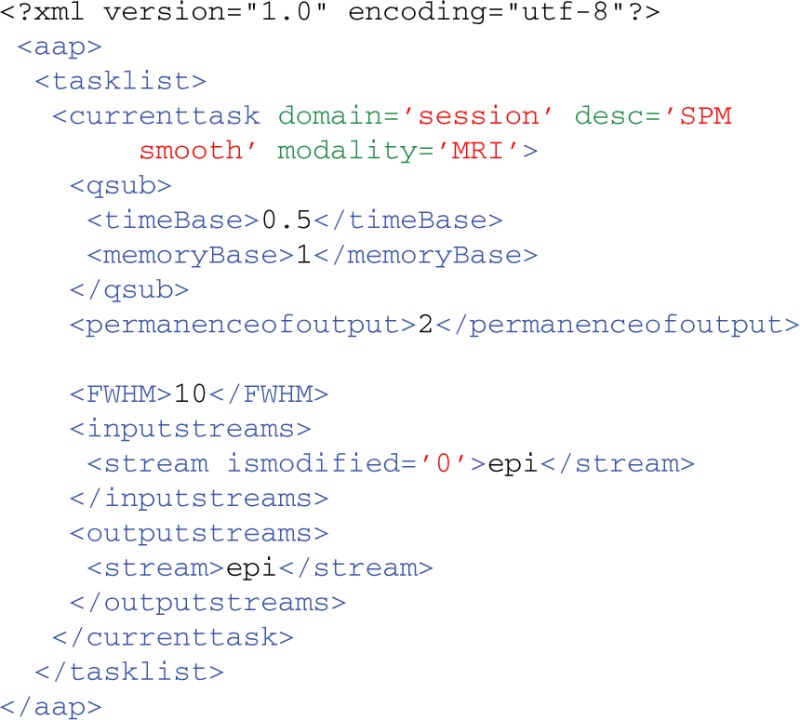



The domain is specified in the attributes of the “currenttask” line, along with a description (which is displayed to the user) and the modality of the data—here “MRI.”

The next two sections are of less focus here. The “qsub” fields are estimates of the resources used by this module, for use by some parallel schedulers. The “permanenceofoutput” field is used by the garbage collection tool to delete less important, intermediate data prior to archiving. Higher numbers correspond to more important data.

More central to the function of this particular module, the “FWHM” field describes a setting of this module—in this case, the full-width half maximum of the smoothing kernel, in millimeters. There is then a description of the sorts of input data (or “streams”) that this module requires, here only “epi” data, and the output data, again just “epi” for this module. The operation of these is discussed more in the next section. The Matlab code for a module implements the function.

### Customizing analysis parameters

In the *aa* user script, the *aarecipe* command sets the initial state of the *aap* structure that describes the analysis:


aap=aarecipe(’aap_parameters_defaults.xml’,
             ’aap_tasklist_typical_fmri.xml’);


The values in this *aap* structure come from three sources:

1. The file *aap_parameters_defaults.xml*, which contains general settings;
2. The tasklist XML file (here *aap_tasklist_typical_fmri.xml*);
3. The XML interface files for each of the modules in the tasklist.

The values returned by the *aarecipe* command are often customized in the user script. Any parameter in *aap* may by modified. Examples are:


aap.acq_details.numdummies=3;
aap.tasksettings.aamod_smooth.FWHM=8;


Alternatively, it is sometimes more convenient to create modified XML files. XML tasklists may set parameters for an individual instance of a module, with syntax like this:

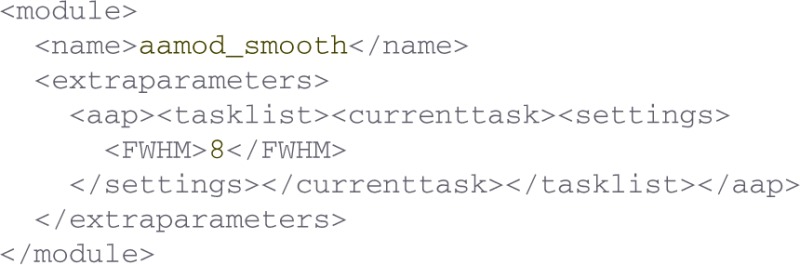



It is also possible to create XML files that inherit the parameters from the standard files, and override a few of them. For example, one can create a site/study/specific version of *aap_parameters_defaults.xml*, such as *aap_parameters_defaults_CBSU.xml* (specific for the MRC Cognition and Brain Sciences Unit):

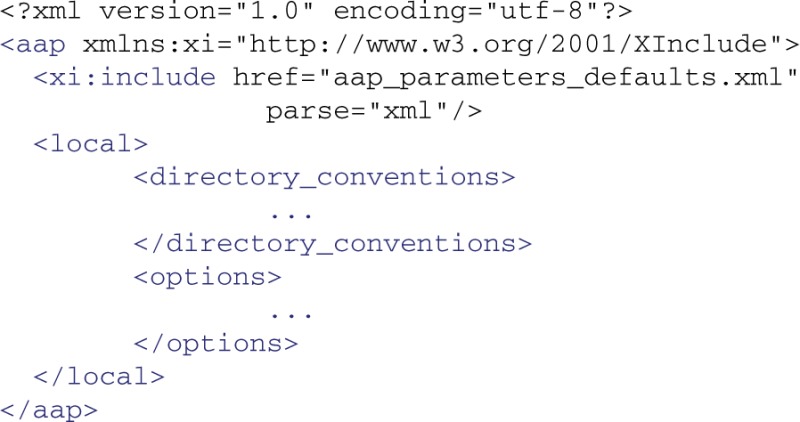



in which most of the settings are imported from *aap_parameters_defaults.xml* using XML Inclusion (http://www.w3.org/TR/xinclude) and only the path-related settings are redefined in the *<local/>* section.

SPM defaults are a special case. These can be modified in the *aap.spm.defaults* structure.

### Specification of statistical models for fMRI

For users who wish to analyze fMRI data with *aa*, a simple set of commands is available for the specification of first-level statistical models. The format is:


aap=aas_addevent(aap,modulename,subject,session,
    eventname,ons,dur,parametric);


where:


modulename=module(e.g.,’aamod_firstlevel_model’)
    for which this event applies
subject=subject for whom this model applies
session=session for which this applies
eventname=name of the stimulus or response event
ons=event onset times (in scans). Does not need
    to be sorted
dur=event durations (in scans), either a single
    element (if all occurrences have the same
    duration) or in order that corresponds to ons
parametric=parametric modulator (optional - can
    omit)


For example,


aap=aas_addevent(aap,’aamod_firstlevel_model’,’^*^’,’^*^’,
   ’VisualStimulus’,[0:15:75],7.5);


specifies that every session of every subject was a block design, with a regressor titled “VisualStimulus” with onsets every 15 scans and a duration of 7.5 scans.

Using the “subject” and “session” fields, customized designs for each subject and/or session may be specified.

A contrast may then be specified with


aap=aas_addcontrast(aap,modulename,subject,format,
    vector,contype,automatic_movesandmeans)


where:


modulename= module (e.g.,’aamod_firstlevel_
contrasts’) for which this contrast applies
subject=subject for whom this model applies
format=format for contrast specification, one of:
  * "sameforallsessions" - vector contains contrast
  to be applied to all sessions
  * "singlesession:[sessionname]" - vector contains
  contrast for just one session, all other sessions
  will be set to 0. [sessionname] should be
  replaced with name of that session.
  * "uniquebysession" - long contrast string that
  separately specifies contrast for every session
contype="T" or "F" (defaults to "T")
automatic_movesandmeans=1 or 0, add means & moves
to contrast automatically?


For example,


aap=aas_addcontrast(aap,’aamod_firstlevel_contrasts’,
    ’*’,’sameforallsessions’,[1 -1]);


to contrast the first vs. the second column of every session in every subject.

If the desired second level model is to run a simple *t*-test for every contrast run in every subject at the first level, then the module *aamod_secondlevel_model* may be added to the tasklist. It does not require customization.

### Streams

All data into and out of an instance of a module are managed by the *aa* engine. Each type of data is referred to as a “stream.” Common streams are “epi,” “structural,” and “dicom_header.” Note that these descriptions are deliberately unspecific about the state of the data—e.g., the data in the *epi* stream may be normalized, or not—as subsequent modules (e.g., first level statistics) often do not need to change their behavior to work on one kind of data or another.

A module's interface (XML file) describes the data streams that it requires wants as an input:


<inputstreams>
    <stream>epi</stream>
</inputstreams>


and what it produces as an output:


<outputstreams>
    <stream>realignment_parameter</stream>
    <stream>meanepi</stream>
    <stream>epi</stream>
</outputstreams>


This information is then used to connect up the pipelines of data from one module to the next. So, for example, if a tasklist contains:


<module><name>aamod_realign</name></module>
<module><name>aamod_tsdiffana</name></module>
<module><name>aamod_slicetiming</name></module>


The module *aamod_slicetiming* requests an *epi* input. The quality control module *aamod_tsdiffana* does not produce an *epi* output, so *aa* looks further back up the tasklist (see Figure 1). It finds that *aamod_realign* produces an *epi* ouput, and so it will pass the *epi* output of *aamod_realign* to *aamod_slicetiming*. This automatic connection of pipelines makes it straightforward to rearrange modules.

A complexity that is largely hidden from the user is that dependencies are calculated at the level of particular instances of a module, and are affected by the domains at which the source and target modules operate. Consider this fragment of a tasklist:


<module><name>aamod_norm_write</name></module>
<module><name>aamod_smooth</name></module>


Both *aamod_norm_write* and *aamod_smooth* operate on the domain of single EPI sessions for single subjects. The instance of the module *aamod_smooth* that processes subject 4, session 2, only needs the data from the instance of the module *aamod_norm_write* that has processed subject 4, session 2, and so only the corresponding data are is passed to the module instance. Furthermore, when executing in parallel, each *aamod_smooth* instance may execute as soon as the corresponding *aamod_norm_write* module has completed, and it does not need to wait for any others to finish. Although transparent to the user, dependencies become more complicated when the domain of a module that is the source of a given stream is different from the domain of a module that is the target of that stream. The restriction that is enforced is that any module may only write data at the level of its domain or lower (i.e., not sideways or above in Figure 2). However, modules may read from levels up toward the trunk, but never sideways.

### The scheduling engine and parallel processing

The *scheduling engine* executes all analyses described within the *aap* structure. The command included in every user script is:


aap=aa_doprocessing(aap);


This executes an *aa* analysis. To do this, it builds a map of all the instances of all the modules that need to be executed, and the data dependencies between them.

To test whether an instance of a module needs to be executed, *aa* looks for a file named *done_aamod_[modulename]_[index]*. This file will be stored in the root directory of the instance: for a session domain module, in the session directory. If it exists, that instance is considered to have been completed, and will not be re-run. The exception to this rule is an earlier module instance in the pipeline needing to be rerun, on which this module instance is dependent. This will cause the *done_* flag to be deleted, and the module will be re-run.

*aa_doprocessing* examines the field *aap.options.wheretoprocess* to decide how to run these modules. If the field has a value “localsingle” it will step through these modules one at a time, in the current Matlab process (as implemented in the object *@aaq_localsingle*). If it has the value “qsub” it will use the parallel computing toolbox component “createTask” to submit a job. If it has the value “condor” it will compile the job and submit it to a condor queuing system, using the shell script specified in *aap.directory_conventions.condor_wrapper*. @*aaq_matlab_pct* uses Matlab's parallel computing toolbox.

Ultimately, regardless of the scheduling mechanism, instances of modules are run by calls to the *aa_doprocessing_onetask* function.

### Branched tasklists

Neuroimaging studies frequently require data to be analyzed in different ways. This might be because there is some uncertainty on the ideal parameters or analysis strategy (for example, whether motion correction should be performed before or after slice timing correction, or what smoothing kernel should be used). Alternatively, it might be because the data are to be analyzed in a number of different ways—with ICA, with conventional univariate fMRI, with MVPA, and with functional connectivity^2^.

Traditionally, these scenarios would probably involve either creating entirely independent pipelines, or processing to the branch point, making a copy of the analyzed data in a different directory, and then taking the new analysis forwards. By contrast, *aa* provides a straightforward way of specifying branched tasklists, as in the following fragment:

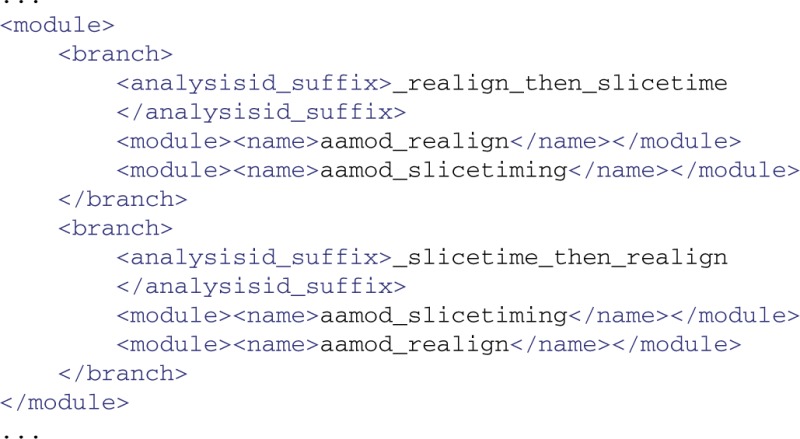



In this command, *<analysisid_suffix>* is included within each branch, so that the two branches get separated into different directories. Although tidy, this is not strictly necessary, as the duplicated modules will be suffixed with different indices—e.g., in the first branch realignment will be output to *aamod_realign_00001* and the second to *aamod_realign_00002*.

### Fully qualified stream references

By default, the input for a stream to a module comes from the last module in the tasklist that outputs that kind of data. Often, this is the desirable behavior. However, sometimes, an explicit earlier reference may be desired. This can be achieved with a fully qualified stream reference comprising *[module-name].[stream-name]* as in this example:

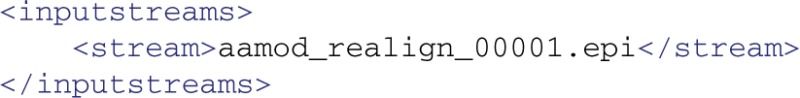



### Adjusting defaults, and site-specific configuration

There are at least two ways a user may customize *aa* for a particular site. One way is to have a site-specific configuration file, conventionally called *aas_localconfig_[sitename]*. This is then inserted into the user script, soon after the recipe command, with the line:


aap=aas_localconfig_[sitename](aap);


Another way is to create a customized *aap_parameters_defaults.xml* file, typically by including the existing *aap_parameters_defaults.xml* file and then overriding some parameters for this local installation, like this:

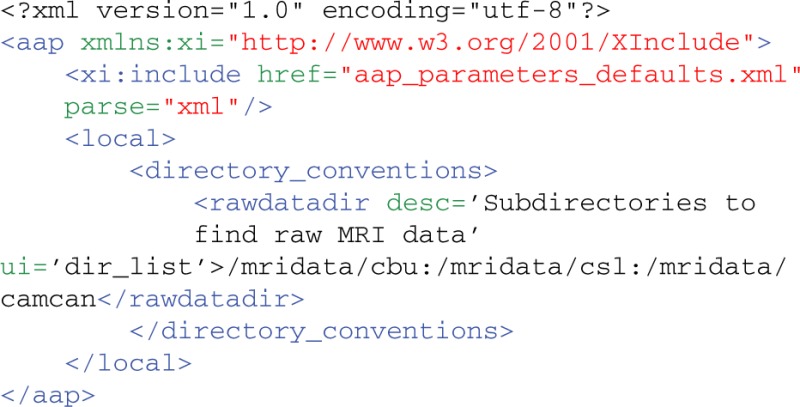



### Input data format

A user must prepare raw data in a form acceptable for input to *aa*. The easiest starting point is typically the raw DICOM data, exported as a set of files from the scanner. One challenge we faced in porting *aa* between sites was that the dumping of the raw data out of DICOM database (PACS) systems led to idiosyncratic filename and directory structures. *aa* will automatically scan the data and structure it into acquisition series for Siemens and GE scanners, provided all of the files from each subject can be isolated into one directory (or a directory with subdirectories). No particular naming convention is required, other than a consistent filename extension for the DICOM files. The DICOM headers are used to organize the files. The system may work also on data from other scanner manufacturers, but we have not tested it.

In a user's tasklist (or later, as a site-specific configuration) the dicomfilter can be set, typically to one of:

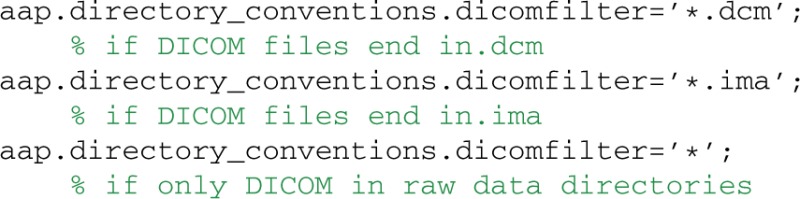



For any tasklist, setting the first main module to *aamod_autoidentifyseries_timtrio* for data from Siemens scanners, or *aamod_autoidentifyseries_ge* with GE scanners, will identify the DICOM files.

Provided researchers use a consistent name for their structural scans, these scans can be automatically identified by setting:


aap.options.autoidentifystructural=true;
aap.directory_conventions.protocol_structural=’MPRAGE’;


The first line requests automatic scanning for the structural (the default), and the second, which protocol should be sought. If a user sometimes acquires more than one structural (for example, if a subject moves) but always stops once they have a good one, it is possible to specify that in this circumstance the last structural is the one to be used:


aap.options.autoidentifystructural_chooselast=true;


A second alternative is to use data already converted into NIfTI format. This is possible, either by using the *aas_addinitialstream* command in the user script, or the *aamod_epifromnifti* module. However, detailed instructions for doing so are beyond the scope of this overview.

### Connecting pipelines

It is often the case that a researcher will want to analyze a subset of data from a larger database, or continue an analysis that exists in a different location (i.e., a remote location). For example, a lab might store and preprocess all their subject MRI data—fMRI, structural images, and diffusion images—on a central server, but one user might want to only analyze the fMRI data from a few subjects on their local machine. *aa* allows a user to easily accomplish this by creating an analysis script that connects to the *aa* pipeline on the central server; the user does not have to manually copy and import any data. The new analysis does not replicate any of the modules or data on the central server, but instead connects the input streams of the local analysis to the data output streams in the remote location. By default, the connection is made to the terminal end of the remote pipeline (i.e., the final instance of each output stream), but the user can easily specify a connection to an earlier stage of processing (e.g., to take the EPI stream before the normalization stage). Furthermore, every time the local analysis is executed, *aa* will check to see if the remote data have changed, and re-run any local modules that depend on those data. The ability to connect pipelines facilitates data sharing within and between labs, promotes good practices for organizing and storing data, reduces data duplication, and simplifies the process of starting new analyses on existing data sets. Detailed examples of this feature are provided in the *aa* documentation.

## Community

*aa* has been used for hundreds of analyses covering many thousands of participants. It is currently supported by a small but active base of coders.

### Brain and mind institute, Western University, London, Canada

Authors Rhodri Cusack, Annika C. Linke, Conor J. Wild and colleagues at the Brain and Mind Institute are actively developing for *aa*, and use it for fMRI, DTI and structural data from a variety of MRI scanners—Siemens 3T (Trio, Prisma), Siemens 7T, and GE 1.5 T (MR450w)—and EEG (EGI, Grass).

### MRC cognition and brain sciences unit, Cambridge, United Kingdom

In addition to authors Tibor Auer and Daniel J. Mitchell a handful of other coders in the Unit also actively participate in developing *aa* modules. In the Unit, *aa* is the backbone of analysing fMRI, DTI, MTR and structural data from Siemens 3T (Trio, Prisma) MRI scanner, Elekta Neuromag Vectorview MEG scanner and Brain Products BrainAmp EEG. New colleagues are introduced to *aa* right from the start by means of workshops, which allow them to perform analysis quite early on. A highlighted project, the Cambridge Centre for Aging and Neuroscience, involving multiple sessions of hundreds of subjects, also employs *aa*, which ensures both high consistency via standardized user scripts and tasklists and high processing speed via parallelization. The Unit also hosts a wiki (http://imaging.mrc-cbu.cam.ac.uk/imaging/AA) complementing the *aa* documentation.

### Donders center for cognitive neuroscience, Nijmegen, the Netherlands

Author Alejandro Vicente-Grabovetsky and colleagues in the Doeller laboratory are actively developing for *aa*, and use it for Siemens 3T and 7T fMRI analyses.

### Washington University in St Louis

Author Jonathan E. Peelle and his laboratory are developing structural and functional MRI analysis for Siemens 3T data.

## Github source control, support, and documentation

The codebase is maintained at: https://github.com/rhodricusack/automaticanalysis.

There are two main branches: the *master* branch, which contains a recent stable release, and the *devel-share* branch, which contains the latest versions of the code published by each of our sites. There are also occasional releases, under “tags,” which contain frozen past versions of the code.

A website (http://automaticanalysis.org) contains the latest documentation for the code, and an issues discussion forum is used to report bugs or ask questions (https://github.com/rhodricusack/automaticanalysis/issues).

## Other design decisions

Our software provides access to most functions of SPM, one of the most commonly used neuroimaging tools worldwide, for analyses such as fMRI modeling and voxel-based morphometry. For several diagnostics in general and DWI analysis we use the well-established FSL functions, and for cortical-surface based measures, Freesurfer.

## Limitations

Every processing approach has limitations, and *aa* is no different. Perhaps the biggest hurdle for novices is the requirement of knowing enough Matlab to organize analyses. The choice of Matlab as a programming language grew out of the origins of *aa* as a pipeline for SPM. There are clearly advantages and disadvantages to this choice. Matlab is widely used in neuroimaging, other areas of neuroscience, engineering and finance, and Matlab programming is a skill that is transferrable to other disciplines. The language provides an enormous library of high-level mathematical functions that are well tested, and in most cases highly optimized. It provides compact and elegant syntax for matrix math. It has a mature integrated-development environment (IDE) including line-by-line debugging, workspace inspection, computation time profiling, and 2D/3D graphics. It is a well-supported product, with regular updates and new features. A disadvantage is that as a commercial product, it comes with substantial costs, and is not open-source, reducing the potential for quality assurance and innovation directly from the community. However, Matlab does come with a compiler, allowing functions to be redistributed freely (but not to be changed), and it has an active user software exchange.

Like most pipelines that serve as interfaces to other tools, *aa* can be a bottleneck: one can only incorporate into a pipeline those tools that are already “wrapped” into *aa*. For example, there are currently no *aa* modules for *AFNI* tools. However, *aa*'s open source nature and its easy extendibility allow the user to implement the corresponding functionality and even to make it available to others.

Another consequence of automated pipelines such as *aa* is that they facilitate the processing of large datasets, in turn producing more data and increasing demands for file storage. Although *aa* attempts to keep only necessary files through garbage collection, analyses can quickly take up large amounts of disk space if not kept in check, which may prove to be a limitation in some contexts.

Finally, there is always the danger when using automated batch analysis pipelines that the researcher might try every possible combination of analysis tools and parameters —so-called “experimenter degrees of freedom”—to obtain the desired results. This is not a new problem in neuroimaging, but *aa* at least provides a way for researchers to keep track of different analysis approaches through branched tasklists and detailed analysis logs.

Despite these possible limitations, we believe that *aa* is successful in balancing the diverse needs of neuroimagers, and facilitating open, reproducible science on datasets of many sizes and complexities.

### Conflict of interest statement

The authors declare that the research was conducted in the absence of any commercial or financial relationships that could be construed as a potential conflict of interest.

## Footnotes

^1^British spellings are used throughout *aa*, reflecting its country of origin.

^2^Of course, care must be taken when trying out multiple analysis options, and exploration is best done on independent data so as not to bias the results. Our point is that there are many instances in which researchers might reasonably want to compare analysis strategies in a systematic way, which *aa* facilitates.
